# The Risk of Reported Cryptosporidiosis in Children Aged <5 Years in Australia is Highest in Very Remote Regions

**DOI:** 10.3390/ijerph120911815

**Published:** 2015-09-18

**Authors:** Aparna Lal, Emily Fearnley, Martyn Kirk

**Affiliations:** National Centre for Epidemiology and Population Health, Building 62, Australian National University, Acton, Canberra 2602, Australia; E-Mails: emily.fearnley@anu.edu.au (E.F.); martyn.kirk@anu.edu.au (M.K.)

**Keywords:** cryptosporidiosis, children, geographic, remote

## Abstract

The incidence of cryptosporidiosis is highest in children <5 years, yet little is known about disease patterns across urban and rural areas of Australia. In this study, we examine whether the risk of reported cryptosporidiosis in children <5 years varies across an urban-rural gradient, after controlling for season and gender. Using Australian data on reported cryptosporidiosis from 2001 to 2012, we spatially linked disease data to an index of geographic remoteness to examine the geographic variation in cryptosporidiosis risk using negative binomial regression. The Incidence Risk Ratio (IRR) of reported cryptosporidiosis was higher in inner regional (IRR 1.4 95% CI 1.2–1.7, *p* < 0.001), and outer regional areas (IRR 2.4 95% CI 2.2–2.9, *p* < 0.001), and in remote (IRR 5.2 95% CI 4.3–6.2, *p* < 0.001) and very remote (IRR 8.2 95% CI 6.9–9.8, *p* < 0.001) areas, compared to major cities. A linear test for trend showed a statistically significant trend with increasing remoteness. Remote communities need to be a priority for future targeted health promotion and disease prevention interventions to reduce cryptosporidiosis in children <5 years.

## 1. Introduction

Cryptosporidiosis, caused by the parasite *Cryptosporidium*, manifests as gastroenteritis and is transmitted by the faecal—oral route [1]. *Cryptosporidium* infection may be spread by contact with infected animals or humans, or consumption of contaminated water and foods [2,3,4,5,6,7]. *Cryptosporidium* spp. oocysts are resistant to high concentrations of chemical disinfectants commonly used to treat water for drinking or recreational use, and the infectious dose required to induce infection is relatively small [8]. The oocysts can persist in the environment for a prolonged time [1]. The low infectious dose and extended infectious capability of *Cryptosporidium* spp. help explain why it is easily transmitted through the environment and how the disease is a challenge to control.

The recent Global Enteric Multi-Centre Study estimated that cryptosporidiosis was associated with a significant increase in the risk of death in children aged 12–23 months in low income countries [9]. In industrialized countries, children <5 years bear the greatest burden of cryptosporidiosis [10,11,12]. While cryptosporidiosis is usually self-limiting, children who are immuno-suppressed and undernourished may experience prolonged cryptosporidial diarrhoea and occasionally die [13]. A recent review found that *Cryptosporidium* spp. was significantly more prevalent in malnourished children 2–5 years of age with diarrhoea compared with non-malnourished children [14]. In Brazil, cryptosporidiosis in 0–2 year olds was also associated with reduced cognition in later life, despite adequate nutrition at a later age [15]. Effective methods to control and treat cryptosporidiosis among children present an ongoing problem in need of attention as it is one of the most common causes of early childhood diarrhoea.

Geographic variation in the distribution and spread of disease can help identify locality specific exposures and risk factors, which may be targeted for disease control. One of the key features in the distribution of cryptosporidiosis is a marked seasonality and distinct age-related variation in reported incidence rates [11,16,17]. Seasonal changes can influence transmission potential, host susceptibility and behaviour, and the resistance of *Cryptosporidium* to changes in ambient physical conditions. While national level patterns across different age groups are commonly reported for cryptosporidiosis, overlooking the geographic variation within these patterns limits our ability to develop targeted interventions to prevent disease.

Cryptosporidiosis, confirmed through laboratory diagnosis has been nationally notifiable to health departments conducting public health surveillance in Australia since 2001. Past studies of cryptosporidiosis in Australia have largely focused on limited geographic locations over long time periods [18,19], or on many specific locations over short time periods (e.g., outbreak investigations) [20,21,22]. Collectively, these studies have shown differences in the regional incidence of cryptosporidiosis, and identified common sources of outbreaks. However, these studies are limited in the information they can provide on disease patterns across the country over long time periods which is useful to highlight the underlying risk factors for disease.

We analyse population-based surveillance data to describe the geographic variation in reported cryptosporidiosis in children <5 years in Australia from 2001 to 2012. We spatially link data on individual illnesses to an index of geographic remoteness to examine the association between remoteness and disease, after controlling for the well-established relationship of cryptosporidiosis with season.

## 2. Methods

### 2.1. Data Sources

We obtained data on all cases of cryptosporidiosis reported during 2001–2012 in Australia to the National Notifiable Diseases Surveillance System (NNDSS). The NNDSS is managed by the Australian Government Department of Health and is overseen by the Communicable Disease Network Australia [23]. Case data obtained included date of reporting, age group (in five year age groups), gender, State/Territory and postcode of residence. Ethical approval for the study was obtained from the Australian National University and the Australian Capital Territory Department of Health prior to data release. A case was defined as detection of *Cryptosporidium* in faeces, as per the national case definition [24].

Population denominator data including age structure were based on the Estimated Resident Population (ERP) produced by the Australian Bureau of Statistics (ABS) for the 2011 National Census [25]. The incidence risk was calculated by dividing the total number of notified cases over the 12-year period with an estimate of the population at risk. Geographical State and Territory and postal area boundaries were obtained from the ABS. Postal area boundaries are produced by the Australian Bureau of Statistics and differ between each census, conducted every five years. This is due to population changes, changes to postal distribution areas and changes to the ABS methodology in defining postal area boundaries. For 2011, the methodology was changed to ensure postal areas matched Statistical Area 1 boundaries. The ABS has not produced correspondence files allowing postal areas from 2001 and 2006 to be matched to 2011 boundaries. Therefore in this paper, data were mapped using the 2011 postal area boundaries. More information on the 2011 postal area boundaries can be found at www.abs.gov.au.

### 2.2. Analysis

National age specific incidence risks of reported cryptosporidiosis were calculated using the 2011 ERPs per 100,000 population. Seasonal patterns were visualized using the total number of notifications by week over the study period aggregated by age groups. As data were available at the postal area level, using the ABS index of remoteness for 2011 [26], each postcode was classified as one of five categories for remoteness to describe reported cases by location. The ABS classification for remoteness designates areas as “major cities”, “inner regional”, “outer regional”, “remote” and “very remote”.

To analyse how seasonal cryptosporidiosis risk may vary across an urban-rural gradient, cases in the <5 year age group were grouped into seasons based on the date of reporting of disease. Season was categorized as summer (December, January, and February weeks 49–52 and 1–9), autumn (March, April, and May, weeks 10–22), winter (June, July, and August, weeks 23–35), and spring (September, October, and November, week 36–48). The variable year was defined as a consecutive 12-month period from 1 January to 31 December.

We used negative binomial regression, adjusted for population size, season and gender to identify associations between the risk of reported cryptosporidiosis and an urban-rural gradient. Model coefficients were exponentiated to produce Incidence Risk Ratios (IRR) with corresponding 95% Confidence Intervals (CI). An IRR was the incidence risk in the category of interest compared to the incidence risk in the reference category. For this analysis, major cities were chosen as the reference category as they consistently had the highest number of reported illnesses. Variables with a *p*-value equal to or less than 0.05 were considered significant. A chi-square test for linear trend was performed to determine if cryptosporidiosis systematically increases or decreases over the range of remoteness categories. Stata v13.0 and Microsoft Excel 2010 were used for data management and analysis including incidence risk calculations and regression models. ArcGISv10.0 was used to spatially link reported illnesses to remoteness category by postcode and visualise the average annual rates of reported illness.

## 3. Results

### 3.1. Time Trends and Seasonality in Cryptosporidiosis Reported in Children <5 Years

**Figure 1:**
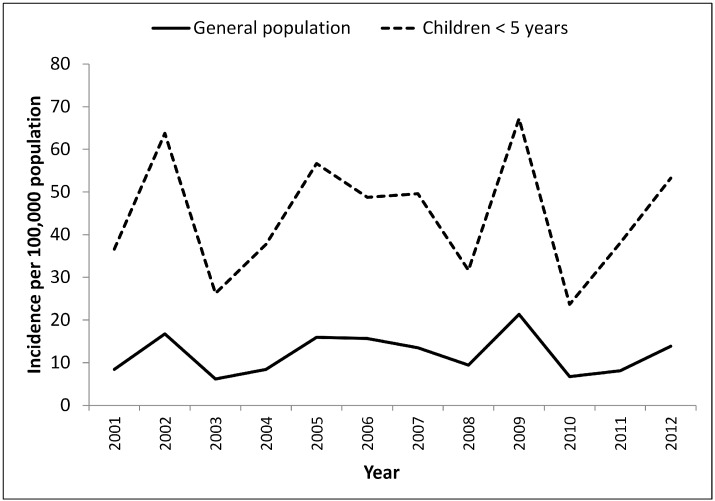
Average annual rates of reported cryptosporidiosis per 100,000 population across Australia in the whole population and in children <5 years, 2001–2012.

From 2001–2012, there were a total of 12,949 cases of cryptosporidiosis reported in children <5 years in Australia. The average annual rate of reported cryptosporidiosis in children <5 years was 105.2 illnesses per 100,000 population compared to the national crude rate of 12.8 illnesses per 100,000 population from 2001 to 2012. Cryptosporidiosis notifications in children <5 years remained consistently higher than the general population and showed a long term trend similar to that of the general population over the 12 year period from 2001 to 2012 (Figure 1). The rate of reported illnesses in children <5 years peaked in 2002, 2005, 2009 and was lower in the years 2003, 2008 and 2010 (Figure 1). The number of illnesses in children <5 years was highest in 2009 with 1840 reported illnesses nationally. The average annual incidence of reported cryptosporidiosis in children <5 years per 100,000 population was 10.2 in the Australian Capital Territory, 11.8 in New South Wales, 82.2 in the Northern Territory, 23.1 in Queensland, 16.6 in South Australia, 19.3 in Tasmania, 12.0 in Victoria, 22.2 in Western Australia. The male to female ratio of reported illnesses in children <5 years was 1.27.

**Figure 2:**
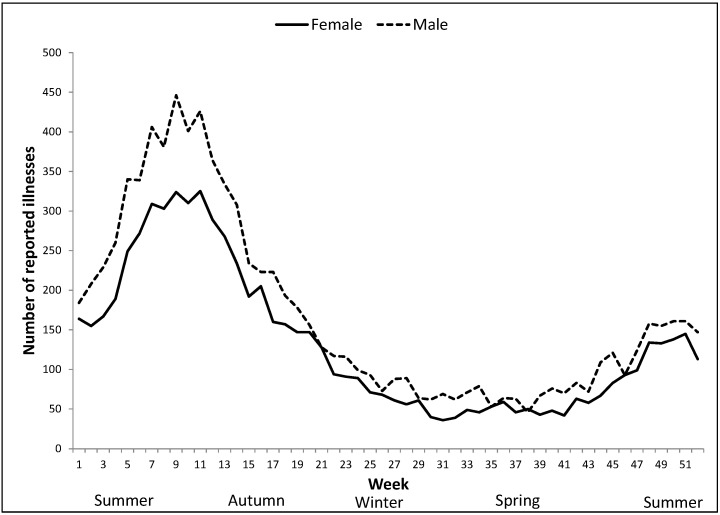
Total weekly number of reported cryptosporidiosis illnesses across Australia for male and female children <5 years, 2001–2012.

Nationally, illness in both male and female children <5 years showed a prominent seasonal pattern. Illnesses peaked towards the end of summer starting in week four (late January) and ending in week 12 (March) (Figure 2). There was also a smaller secondary upward trend in children <5 years aged children reporting illnesses in spring, from week 41 (October) onwards to week 49–50, followed by a decrease in week 51 (late December) (Figure 2).

### 3.2. Geographic Variation in Disease Risk

**Figure 3:**
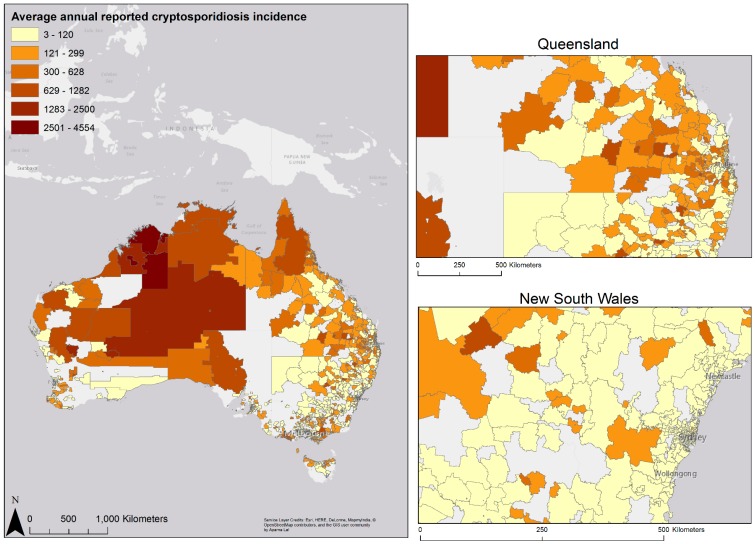
Average annual incidence of reported cryptosporidiosis in children <5 years by 2011 Postal area boundaries.

The rates of reported cryptosporidiosis per 100,000 population in children <5 years varied by remoteness category. In major cities the average annual incidence risk was 12.2 illnesses per 100,000 population, 16.0 illnesses per 100,000 population in inner regional areas, 29.6 illnesses per 100,000 population in outer regional areas, 62.6 illnesses per 100,000 population in remote areas and 89.1 illnesses per 100,000 population in very remote areas (Table 1). Figure 3 shows the average annual rates of reported cryptosporidiosis incidence across the 2011 postal areas in Australia with the highest rates in northern, remote parts of Western Australia, and low rates in major cities like Brisbane and Sydney (Figure 3 and Figure S1).

A negative binomial regression model of cryptosporidiosis risk by remoteness after controlling for season and gender showed significant differences in the risk of reported cryptosporidiosis across an urban-rural gradient (Table 1). Compared to major cities, the IRR of cryptosporidiosis was higher in all other remoteness categories: inner regional (IRR 1.4 95% CI 1.2–1.7, p < 0.001), outer regional (IRR 2.4 95% CI 2.2–2.9, *p* < 0.001), remote (IRR 5.2 95% CI 4.3–6.2, *p* < 0.001) and very remote areas (IRR 8.2 95% CI 6.9–9.8, *p* < 0.001). The test for linear trend across the remoteness categories was statistically significant (*p* < 0.001).

**Table 1:** Negative binomial regression model results of incidence risk of cryptosporidiosis reported in Australian children <5 years across an urban-rural gradient, controlling for season, population size and gender, 2001–2012.

	Number of Reported Illnesses	Average Annual Incidence Risk per 100,000 Population	Incidence Risk Ratio	95% CI^+^	*p*-Value
**Regions**					
Major cities	2466	12.2	1.0		
Inner regional	5355	16.0	1.4	1.2–1.7	<0.001
Outer regional	2536	29.6	2.4	2.0–2.9	<0.001
Remote areas	1029	62.6	5.2	4.3–6.2	<0.001
Very remote	1563	89.1	8.2	6.9–9.8	<0.001
**Gender**					
Male	7243	19.1	1.0		
Female	5706	17.1	0.9	0.8–1.0	>0.1
**Season**					
Summer	4727	22.0	1.0		
Autumn	5077	21.1	0.7	0.6–0.8	<0.001
Winter	1511	11.1	0.3	0.3–0.4	<0.001
Spring	1634	13.4	0.4	0.3–0.5	<0.001

^+^Confidence interval.

## 4. Discussion

The risk of reported cryptosporidiosis in children <5 years is greatest in very remote regions in Australia, compared to major cities. We show a dose-response relationship between disease risk and living in remote areas. There is a clear need to improve understanding of the epidemiology of cryptosporidiosis in remote areas of Australia to better prioritise interventions for diarrhoeal illness.

The association of cryptosporidiosis with remote living is in agreement with studies reporting a positive relationship between reported cryptosporidiosis and rural living. Livestock, wild and companion animals have been implicated as potential reservoirs of infection in these areas. Livestock are an important reservoir for *Cryptosporidium* [27]. Direct contact between young livestock and children living on farms [28], children frequently visiting a farm [29] or consumption of raw milk may be risk factors for infection [22,30]. Exposure to *Cryptosporidium* positive wild-bird droppings [31] and wild marsupials, deer, rabbits and rodents may also result in human disease, although the public health importance of these reservoirs needs to be clarified [32,33,34]. Dogs and cats are also commonly colonized by *Cryptosporidium* [35]. Although pets are not considered a major pathway for transmission of strains capable of causing human infection [36], this has not been explored in remote areas where the density and contact with domestic animals may be high.

It is likely that some of the regional patterns we observed in reported illnesses are due to different *Cryptosporidium* species [37,38], reflecting distinct reservoirs of infection. In Spain, for the livestock adapted strain of *Cryptosporidium* in humans (*C*. *parvum*), incidence peaked during summer and was mainly confined to rural areas, while for the human adapted strain (*C*. *hominis*), infections were more common in autumn with a more widespread geographical distribution [39]. A similar species specific seasonal and geographic pattern has also been reported in England and Wales [5]. In New Zealand, in addition to the strain specific seasonal patterns documented elsewhere, *C. hominis* was dominant in urban regions, whereas *C. parvum* was prevalent in rural New Zealand [38]. We were unable to analyse data based on the species of *Cryptosporidium* due to missing data on species isolated and the data on speciation from routine pathology reports may be unreliable due to different methods for identification across States and Territories in Australia.

The high rates of reported cryptosporidiosis in remote areas may be related to the high rates of diarrhoeal illness in Aboriginal and Torres Strait Islander children in these areas [40]. In Canada, remote Indigenous communities have a higher burden of zoonotic parasitic diseases [41]. For Indigenous Status, the incompleteness of reporting is a major issue, due to State and Territory wide discrepancies in completeness of reporting. For example, while the Northern Territory had Aboriginal and Torres Strait Islander (ATSI) status reported for 98% of all cryptosporidiosis cases reported from 2001–2012, data from Queensland was only 44% complete with 56% of records with a blank field for ATSI status. Due to incomplete reporting of Aboriginal and Torres Strait Islander status in the disease notification data and the absence of reliable strain typing data, we were unable to address these factors in our study. However, childhood diarrhoea has been identified as a key health outcome that needs to be addressed to reduce health disparities in remote Australian regions [42]. Our results emphasize the need for targeted health promotion and education programs tailored to reduce childhood diarrhoea in remote communities.

In addition to studies that determine the public health importance of infection reservoirs in remote areas, a detailed investigation of the potential transmission pathways in these areas also needs to be carried out. Faecal contamination of drinking water sources has been implicated as the source of several outbreaks of cryptosporidiosis elsewhere [43]. In remote Australia, the lack of adequate potable drinking water is a major health issue [44] and may partly be responsible for the high cryptosporidiosis rates in these regions. Increased rainfall in summer in the northern tropical regions of Australia may increase gastroenteritis in areas using untreated water for drinking, while seasonal rainfall may not influence illness in areas with treated municipal water supplies [45]. There is also a growing body of evidence to suggest that recreational water activities are a major transmission route for cryptosporidiosis. As swimming has been identified as a risk factor for cryptosporidiosis in several studies [46,47], the risk with remote living may be indicative of increased recreational water activities in summer [20]. In Australia, a summer outbreak of cryptosporidiosis was linked to a public swimming pool in a remote region [20]. We found a summer to early autumn peak in cryptosporidiosis incidence, similar to that previously reported in the United States [48], and a general increase in infectious gastroenteritis in Australia over summer [49]. Exposure to untreated drinking water sources or recreational activities could partly explain this higher disease risk in remote areas.

To date, there are few studies that have focussed on the importance of person-to-person transmission in remote areas. Outbreaks of cryptosporidiosis in child care settings through person-to-person transmission have been reported [50]. A study in Spain found higher prevalence of *Cryptosporidium* in children during the winter and spring months, with authors attributing this seasonal pattern to attendance at day care centres [51]. Person-to-person transmission of cryptosporidiosis in hospital settings has also been shown [52]. However, in remote areas, the importance of person-to-person transmission is a knowledge gap that remains to be addressed. Remote living may in some cases, be an indicator for substandard housing conditions which can lead to person-to-person infection spread. For example, inadequate sanitation and hygiene infrastructure can exacerbate transmission in remote communities [53]. In remote Australian communities, it has been suggested that household crowding straining existing washing and sanitary infrastructure partly contributes to the high prevalence of intestinal parasites in children [54]. In young children, unintended ingestion of faecal pathogens following hand-to-mouth contact is an important risk factor for diarrhoea [55], and person-to-person transmission is likely to be an important mode of spread for *Cryptosporidium* spp. in remote settings. Targeted health education and promotion programs [56], alongside improvements in housing infrastructure in remote areas [57] is likely to be an effective method to reduce cryptosporidiosis, and diarrhoeal disease in children generally.

Our study has some general limitations. First, population based laboratory surveillance systems generally capture a fraction of all illnesses occurring in the community for several reasons. There might be differences in underreporting across age groups, as children and the elderly are more likely to visit a physician and get stool samples tested. By concentrating on children <5 years we minimize this bias that may affect comparisons across age groups. Second, there might be geographic variation in the reporting of cryptosporidiosis due to differences in health care providers’ accessibility and clinical differences between cases across regions due to access to health care. Our finding of higher risk in remote and very remote areas compared to major cities suggests that these differences were likely to be minor. Third, seasonality in reporting may contribute to the later spring and summer peak in notifications [58]. The small downward trend in the last two weeks of December may partly be a surveillance artefact that is attributable to limited laboratory staff and the reduced likelihood of people visiting doctors over the Christmas holiday period. It would be interesting to see whether the higher number of notifications in late spring reflects testing practices or represents an actual increase in cryptosporidiosis incidence. However, this is unlikely to be a deciding factor since seasonality in cryptosporidiosis has been observed in several different countries [59]. We acknowledge that the “traditional” aggregation of months into seasons may mask finer scale temporal patterns. Finally, our study would have benefited from an analytical stratification by *Cryptosporidium* spp. and Indigenous status.

## 5. Conclusions

The burden of reported cryptosporidiosis in children <5 years in Australia is disproportionately high in remote communities. Our results show that the risk of reported cryptosporidiosis is significantly higher in remote and very remote areas, compared to the major cities, after controlling for season and gender. We recommend that children residing in remote areas be targeted for health promotion and prevention and control programs designed to decrease the incidence of cryptosporidiosis. Identifying the specific factors (water sources, environmental exposures, social and cultural determinants, and transmission routes) that underlie these regional differences will help tailor interventions to reduce cryptosporidiosis in children <5 years in Australia.

## Supplementary Files

Supplementary File 1
