# Supplementary material for: The Risk of Reported Cryptosporidiosis in Children Aged <5 Years in Australia is Highest in Very Remote Regions
Source: Int J Environ Res Public Health. 2015 Sep 18;12(9):11815–28. doi: 10.3390/ijerph120911815 (PMC4586709; doi:10.3390/ijerph120911815)
Supplement: Supplementary File 1 [file ijerph-12-11815-s001.pdf]

Supplementary Information

The Risk of Reported Cryptosporidiosis in Children Aged <5 Years in Australia is Highest in Very Remote Regions

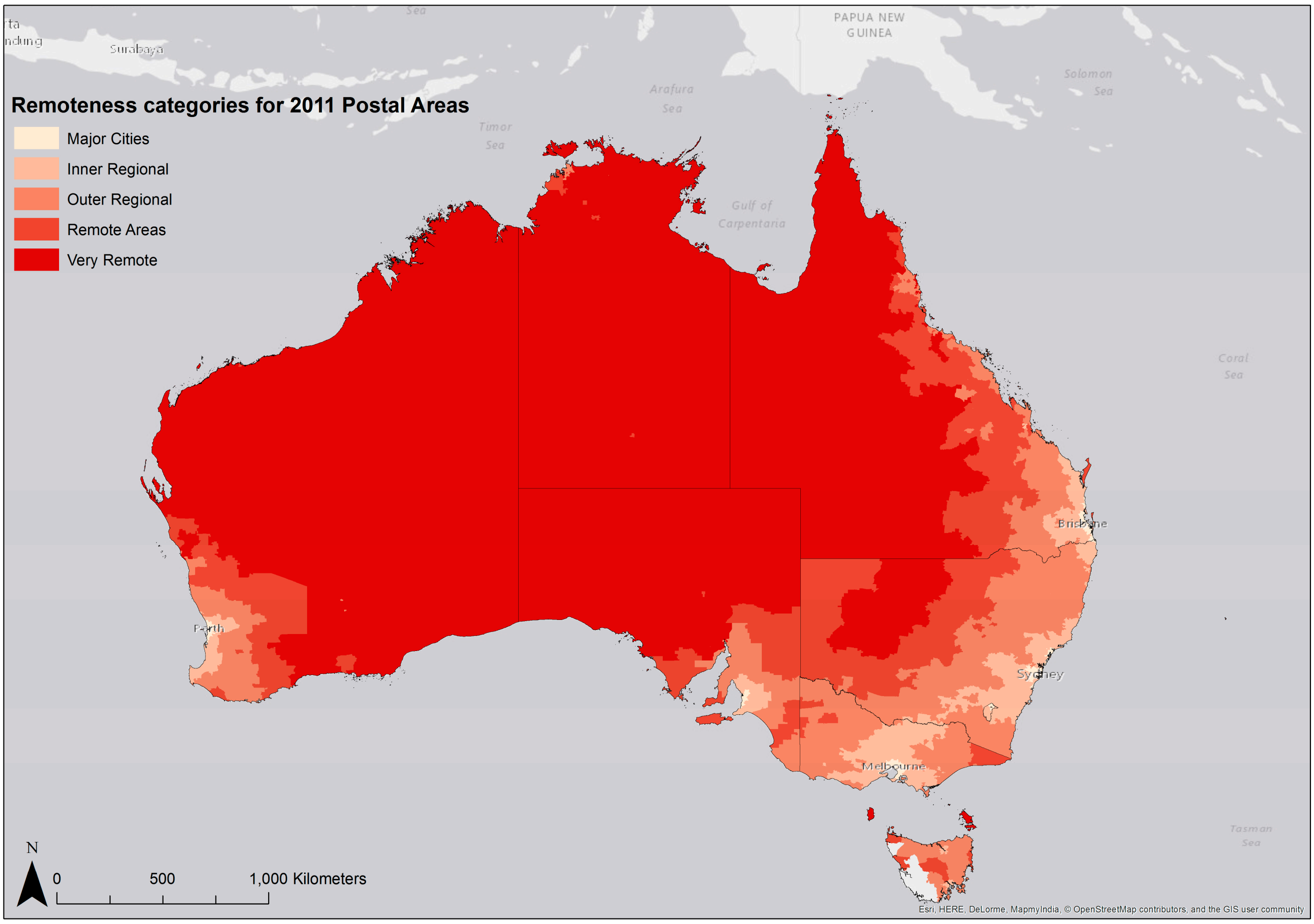

Figure S1. Remoteness categories by 2011 Postal Areas across Australia.

© 2015 by the authors; licensee MDPI, Basel, Switzerland. This article is an open access article distributed under the terms and conditions of the Creative Commons Attribution license (http://creativecommon.org/licenses/by/4.0/).
